# The Role of Elacridar, a P-gp Inhibitor, in the Re-Sensitization of PAC-Resistant Ovarian Cancer Cell Lines to Cytotoxic Drugs in 2D and 3D Cell Culture Models

**DOI:** 10.3390/ijms26031124

**Published:** 2025-01-28

**Authors:** Piotr Stasiak, Justyna Sopel, Julia Maria Lipowicz, Agnieszka Anna Rawłuszko-Wieczorek, Jan Korbecki, Radosław Januchowski

**Affiliations:** 1 Institute of Biological Sciences, University of Zielona Góra, 65-417 Zielona Góra, Poland; 2 The Doctoral School of Exact and Technical Sciences, University of Zielona Góra, 65-417 Zielona Góra, Poland; 3 Institute of Health Sciences, Collegium Medicum, University of Zielona Góra, 65-417 Zielona Góra, Poland; j.sopel@inz.uz.zgora.pl (J.S.); j.korbecki@inz.uz.zgora.pl (J.K.); r.januchowski@inz.uz.zgora.pl (R.J.); 4 Department of Histology and Embryology, Doctoral School, Poznan University of Medical Sciences, 61-701 Poznań, Poland; julia.lipowicz@student.ump.edu.pl; 5 Department of Histology and Embryology, Poznan University of Medical Sciences, 61-701 Poznań, Poland; arawluszko@ump.edu.pl

**Keywords:** elacridar, multidrug resistance (MDR), P-glycoprotein (P-gp), chemotherapy resistance, molecular inhibitors, three-dimensional (3D) cell culture, ABC transporters activity, ovarian cancer

## Abstract

Chemotherapy resistance is a significant barrier to effective cancer treatment. A key mechanism of resistance at the single-cell level is the overexpression of drug transporters in the ABC family, particularly P-glycoprotein (P-gp), which leads to multidrug resistance (MDR). Inhibitors of these transporters can help re-sensitize cancer cells to chemotherapeutics. This study evaluated elacridar (GG918 and GF120918), a potent third-generation P-gp inhibitor, for its ability to reverse MDR in paclitaxel (PAC)-resistant ovarian cancer cell lines. Sensitive and PAC-resistant cells were cultured in two-dimensional (2D) and three-dimensional (3D) models. *MDR1* gene expression was analyzed using Q-PCR, and P-gp protein expression was examined via Western blot and immunofluorescence. Drug sensitivity was evaluated with MTT assays, and P-gp activity was analyzed by flow cytometry and fluorescence microscopy. Elacridar effectively inhibited P-gp activity and increased sensitivity to PAC and doxorubicin (DOX) in 2D cultures but not cisplatin (CIS). In 3D spheroids, P-gp activity inhibition was observed via Calcein-AM staining. However, no re-sensitization to PAC occurred and limited improvement was observed for DOX. These findings suggest that elacridar effectively inhibits P-gp in both 2D and 3D conditions. However, its ability to overcome drug resistance in 3D models is limited, highlighting the complexity of tissue-specific resistance mechanisms.

## 1. Introduction

The problem of chemotherapy resistance in cancer poses a major obstacle in the treatment of this disease [1]. Principally, two types of drug-resistance mechanisms can be distinguished: cell-specific drug resistance mechanisms (increased drug inactivation, increased DNA repair, or increased drug elimination from cancer cells) and tissue-specific drug resistance mechanisms related to tumor microenvironment (a high density of cells in the tumor and overexpression of extracellular matrix proteins). In practice, both resistance mechanisms play together in tumors, leading to chemotherapy resistance [2].

The most important mechanism of resistance at the cellular level is known as multidrug resistance (MDR). MDR is the phenomenon in which cancer cells treated with chemotherapy develop cross-resistance to many drugs, even those of very different chemical structures [3]. The main cause of MDR is the overexpression of ATP-binding cassette (ABC) proteins—transmembrane ATP-ases capable of the active removal of various types of drugs [4].

Glycoprotein P (P-gp, MDR1) is a member of the eukaryotic ABC superfamily of membrane transporters [5]. It is the most well-understood mammalian ABC protein regarding its structure and mechanism of action [6]. P-gp was first described in 1976 after being discovered in drug-resistant ovary cells of the Chinese hamster. It was established to be a glycoprotein, and since it appeared exclusively in cells with altered drug permeability it was named the “permeability glycoprotein”, shortened as P glycoprotein or P-gp [7]. The *MDR1* gene encoding P-gp is composed of 29 exons and is localized in chromosome 7q21.12 [8]. P-gp comprises 1280 amino acids and is classified as a surface ATP-ase. The molecular mass of the protein is 140–150 kDa [9] and increases to 170 kDa after N-glycosylation on amino acids 91, 94, and 99 [9,10]. The protein has 12 transmembrane domains (TMD), two nucleotide-binding domains (NBD), and two ATP-binding domains that are located on the cytoplasmic intracellular part of the protein; however, it does not have a well-defined ligand-binding pocket [11].

Physiologically, in normal human tissues, the P-gp is expressed in many epithelial cells [12], including those with secretory and excretory functions [13]. Several examples include epithelium lining the trachea, major bronchi, biliary ducts, mammary glands, prostate, kidneys, intestine and brain–blood barrier [12,14]. The function of P-gp is to prevent the entrance of xenobiotics and harmful chemicals to the cells [11], as well as the biliary excretion of various compounds of endogenous and exogenous origin taking place in the liver [13]. The strong expression of P-gp is particularly important to the function of blood–tissue barrier sites, for example on endothelial cells of capillary blood vessels supplying the brain (at the blood–brain barrier) [12]. The low oral bioavailability of some drugs is the result of the intestinal expression of P-glycoprotein (P-gp) [14].

Unfortunately, high P-gp expression is often observed throughout various kinds of cancer, including breast, colon, lung, and ovarian cancer, and many more, resulting in MDR [15]. In cancers, P-gp overexpression can be either inherent or acquired following drug treatment [5].

The broad substrate specificity of P-gp is responsible for its role in MDR. P-gp is capable of the ATP-dependent removal of various compounds from the cell, including important chemotherapeutics, such as actinomycin D, daunorubicin, doxorubicin (DOX), epirubicin, etoposide, mitomycin C, mitoxantrone, paclitaxel (PAC), topotecan, vinblastine and vincristine [11].

Among many substrates of P-gp, there are two highly used cytotoxic drugs: PAC and DOX [3]. PAC, a member of taxanes, is a drug that stabilizes microtubules, preventing cell mitosis [16]. It is prescribed for the treatment of ovarian, breast, and lung cancer [17]. In ovarian cancer, PAC serves as a first-line treatment drug [18]. DOX, an anthracycline, intercalates between DNA’s double helix base pairs resulting in the inhibition of DNA replication and transcription [19]. It also inhibits the function of DNA-associated enzymes, such as topoisomerase II and inhibits the process of replication [20]. It is used to treat breast cancer, carcinomas, hematological cancer and sarcomas [21]. It is also administered as a part of the second-line treatment of ovarian cancer [22].

Resistant cells can actively remove cytotoxic agents, and as a result, the therapeutic concentration of the drugs is never achieved. Generally, heightened P-gp expression is associated with poor prognosis for cancer patients [23]. Therefore, there have been many attempts to develop compounds to inhibit P-gp function or compete with chemotherapeutics for P-gp transport [5].

An approach to combat MDR involves the co-treatment of cancer cells with a mixture of cytotoxic drugs and inhibitors of ABC proteins [24]. As a result of ABC protein inhibition, the accumulation of therapeutics inside cancer cells does achieve the required concentration for their cytotoxic function [5]. Depending on their specificity and side effects, molecular inhibitors can be divided into generations, most notably, first, second, and third generation [15]. First-generation inhibitors comprise compounds that were found to inhibit certain protein functions, but were not synthesized specifically for that purpose. They exhibit low specificity and interact with other proteins, often leading to unpredictable side effects. Second-generation inhibitors are molecules modified to have improved specificity and potency compared to the first-generation inhibitors. Third-generation inhibitors are compounds synthesized specifically for the optimal inhibition of the protein in question [11]. Some authors also propose distinguishing so-called fourth-generation inhibitors, which comprise natural compounds capable of limited influence on P-gp activity and cancer growth, including capsaicin, curcumin, limonin, quercetin, and piperine [25]. The most important P-gp inhibitors have been listed in Table 1.

The first known ABC inhibitor was verapamil, a drug used in the treatment of arterial hypertension and a first-generation compound that blocks calcium channels [26]. In the case of second-generation P-gp inhibitors, these compounds often inhibit CYPA4 enzyme and other ABC transporters [27]. Among third-generation inhibitors of P-gp we can distinguish elacridar, one of the most potent P-gp inhibitors [28].

Elacridar, also known as GG918, GF120918, or N-{4-[2-(1,2,3,4-tetrahydro-6,7-dimethoxy-2-isoquinolinyl)-ethyl]-phenyl}-9,10-dihydro-5-methoxy-9-oxo-4-acridine carboxamide is a compound capable of P-gp inhibition [29]. It is a derivative of acridone carboxamide, a tricyclic acridine-based drug used in chemotherapy [30]. Elacridar is a synthetic compound, first discovered by Glaxo (France) during a research program aimed at identifying new inhibitors of mammalian P-gp to overcome the MDR phenotype [30,31]. Its molecular weight is 563.64 g/mol. It has been proven to compete with [3H]azidopine for a common binding site at P-glycoprotein [15,30], with the site itself being most likely located within the transmembrane domains [32]. Although it causes a strong inhibition of P-gp, it is not P-gp specific, as it was also proven to inhibit another important ABC transporter’s protein function, that of breast cancer resistance protein (BCRP) [28].

Elacridar has been studied extensively as a factor capable of re-sensitizing cancer cells to drug treatment [33]. The treatment of a highly resistant leukemic cell line with elacridar resulted in sensitization to daunorubicin and mitoxantrone [34]. In a hepatoblastoma cell line with significantly enhanced *MDR1* expression, elacridar increased cellular response to doxorubicin [35]. In drug-resistant prostate cancer cell lines with positive P-gp expression, elacridar was able to reverse docetaxel resistance [36]. In a P-gp expressing ovarian cancer cell line, the addition of elacridar considerably reversed resistance to all of the P-gp substrates tested [37]. Moreover, elacridar increased topotecan effectivity in several BCRP-overexpressing cell lines, including an ovarian cancer cell line and canine kidney cell culture [38].

Elacridar was tested in preclinical studies in vivo on mice, rats, dogs, and monkeys to evaluate pharmacokinetic parameters and plasma protein binding, demonstrating good absorption, no effect on P450 enzymes, and potential as a P-gp inhibitor in pre-clinical species [39]. Experiments on mice proved that the oral co-administration of the taxanes with elacridar increased PAC and docetaxel concentration in plasma [40]. It was observed that the co-administration of topotecan with elacridar increases the bioavailability of topotecan in mice [41].

In xenograft experiments, elacridar was able to reverse the resistance of P388/DOX tumors to DOX [15]. In tumors created by hepatocellular carcinoma (HCC) cell insertion in mice, elacridar administered in combination with levatinib caused a significant antitumor effect in comparison to levantinib alone [42].

Elacridar has also been tested in clinical trials. A limited number of cancer patients with solid tumors were treated with the combination of DOX and elacridar and experienced significantly increased exposure to doxorubicinol (a metabolite of DOX) in comparison to those treated with DOX only [43]. Moreover, it was established that elacridar significantly increases the systemic exposure to oral PAC in cancer patients [14]. Several clinical studies that were carried out on cancer patients established that elacridar increases the bioavailability of topotecan, with neutropenia being the most common side effect of the inhibitor [44,45].

The 2D cell culture model is perfect for testing cell-specific mechanisms of drug resistance like MDR; however, such a model does not reflect the complicated tumor structure, with a dense cellular structure and the overexpression of ECM molecules that limits drug diffusion [46]. The 3D cell culture model, although not perfect, is much more similar to real tumor conditions. The 3D model of spheroids formation in non-adherent conditions used in our research is an attempt to simulate tissue-specific mechanisms that are present in a cancer tumor environment [47]. Our previous experiments conducted with a 3D model prove that cells grown as spheroids exhibit a higher resistance to chemotherapeutics than the same cells grown as a monolayer [48,49].

A model of ovarian cancer, the most lethal gynecological malignancy, was used to study the effects of elacridar on P-gp protein inhibition. Although most ovarian cancer cases are sensitive to chemotherapy at first, the tumor usually develops drug resistance in response to treatment [50]. We used the drug-sensitive A2780 ovarian cancer cell line, as well as previously derived PAC-resistant ovarian cancer cell lines A2780PR1 and A2780PR2 [51]. To better understand the role of elacridar in the breakdown of resistance to cytotoxic drugs, we performed our experiments on PAC-sensitive and PAC-resistant cell lines in both 2D and 3D cell culture conditions. The hypothesis of our study is that elacridar, a third-generation P-gp inhibitor, can effectively inhibit P-gp activity and reverse multidrug resistance in ovarian cancer cell lines, thereby enhancing sensitivity to chemotherapeutics in vitro.

This study is novel, as to our knowledge, it is the first to evaluate the efficacy of elacridar in overcoming MDR in this unique, dual model of resistance comprising a 2D monolayer and 3D spheroid cultures. While the role of P-gp in mediating MDR has been well-documented in 2D cultures, the impact of P-gp inhibitors on drug resistance in 3D spheroids, which more closely mimic tumor environment, remains poorly understood. This study provides a comprehensive analysis of P-gp activity, expression, and its inhibition under physiologically relevant conditions.

## 2. Results

### 2.1. Characterisation of Drug Resistance in A2780 Cell Line and PAC-Resistant Cell Lines

To study the effect of elacridar on P-gp activity and resistance to cytotoxic drugs, firstly, we characterized the cell lines used in our study. Since we used a drug-sensitive A2780 cell line and its two PAC-resistant sublines, we started our analysis by determining the level of resistance in A2780PR1 and A2780PR2 cell lines through testing cell response to PAC. The A2780 cell line showed sensitivity to PAC, with an IC_50_ value of 2.52 ng/mL. However, for both A2780PR1 and A2780PR2 cell lines, we observed a high increase in resistance to PAC. In the A2780PR1 cell line, we observed a 307-fold increase in IC_50_ value in comparison to the A2780 cell line (IC_50_ = 755 ng/mL vs. IC_50_ = 2.52 ng/mL). Even greater, a 781-fold increase in resistance to PAC was observed in the A2780PR2 cell line (IC_50_ = 1970 ng/mL vs. IC_50_ = 2.52 ng/mL, respectively) (Table 2).

DOX was another cytotoxic drug used in our study. The IC_50_ value for this drug in the sensitive cell line was 22.7 ng/mL. However, in resistant cell lines, the DOX IC_50_ value increased to 2033 ng/mL (A2780PR1) and 6292 ng/mL (A2780PR2), resulting in a 78-fold and 278-fold increase in resistance to DOX, respectively (Table 2).

As CIS is a most important drug used in ovarian cancer chemotherapy, we also compared response to this drug in the investigated cell lines. All tested lines exhibited a high level of CIS-resistance. The determined IC_50_ value for the A2780 cell line was 3763 ng/mL, and for the A2780PR1 and A2780PR2 cell lines it was 7915 ng/mL and 6096 ng/mL, respectively, which results in 2.1-fold (A2780PR1) and 1.62-fold (A2780PR2) higher resistance than that of the sensitive cell line (Table 2).

### 2.2. Analysis of MDR1 Gene and P-gp Protein Expression in Investigated Cell Lines

**Figure 1:**
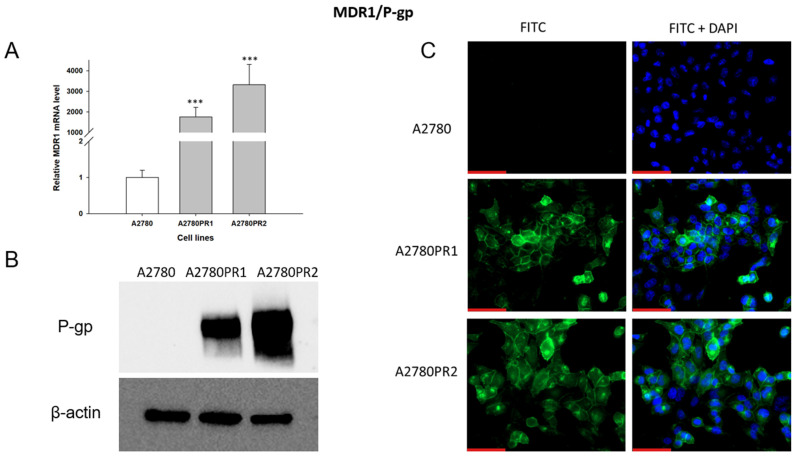
(**A**) Expression analysis of *MDR1* transcript (Q-PCR) in the A2780 and PAC-resistant cell sublines. The figure presents the relative gene expression in the resistant cell lines (gray bars) with respect to the expression in the sensitive cell line (white bar), which has been assigned a value of one. The values were considered significant at *** *p* < 0.001. (**B**) P-gp protein expression analysis in the A2780 and PAC-resistant cell lines grown as a monolayer. The cellular proteins were separated using 7% PAGE and transferred to the PVDF membrane, which was then immunoblotted with either primary Ab or HRP-conjugated secondary Ab. A primary anti-β Ab was used as a loading control for the cell lysates. (**C**) Immunofluorescence visualization of P-gp expression in the A2780 and PAC-resistant cell sublines. P-gp was detected using the anti-P-gp antibody and Alexa Fluor^®^488-conjugated secondary antibody (green). To visualize the cell nuclei, the cells were mounted with a DAPI-containing mounting medium (blue). Objective 40×. Scale bar = 50 μm.

As PAC-resistance is very often associated with the overexpression of the *MDR1* gene and P-gp protein, we started our analysis by determining the expression of MDR1/P-gp in the PAC-resistant and PAC-sensitive cell lines. To characterize the expression of the *MDR1* gene, we analyzed its mRNA expression with the Q-PCR method (Figure 1A). We observed a statistically significant increase in *MDR1* gene expression in both resistant cell lines (*p* < 0.001). The expression of the *MDR1* gene in the A2780PR1 and A2780PR2 cell lines was approximately 2000-fold and 3000-fold higher, respectively, than in the sensitive cell line.

The *p*-gp protein level was confirmed by Western blot analysis (Figure 1B). In the A2780 cell lysates, we did not observe P-gp protein expression. In both PAC-resistant cell lines we observed strong bands corresponding to those of the P-gp protein, with stronger band intensity in the A2780PR2 cell line, a result consistent with the level of *MDR1* gene expression determined for this cell line.

To confirm the presence of P-gp protein in PAC-resistant lines, we also performed immunofluorescence analysis (Figure 1C). These studies confirmed the presence of P-gp protein in PAC-resistant cell lines, with a clear localization of P-gp protein in the cell membrane. However, we did not observe any presence of P-gp protein in the sensitive cell line.

### 2.3. Analysis of P-gp Activity

**Figure 2:**
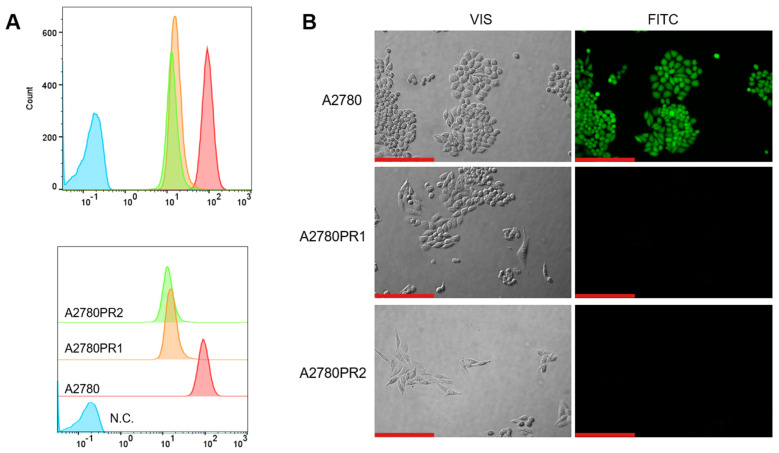
(**A**) Flow cytometry analysis of the intracellular accumulation of Rho123 in drug-sensitive and PAC-resistant cell lines. The diagrams of fluorescence intensity each show the fluorescence intensity of the sensitive cell line A2780 (red) and corresponding resistant cell lines A2780PR1 (orange) and A2780PR2 (green). N.C.—negative control, without Rh123 (blue). (**B**) Inverted fluorescence microscopy analysis of Calcein-AM accumulation (green) in the drug-sensitive A2780 cell line and corresponding A2780PR1 and A2780PR2 cell lines. Objective 20×. Scale bar = 200 μm.

To study the transport activity of the P-gp protein in sensitive and PAC-resistant cell lines, the intracellular ability to accumulate Rho123 and Calcein-AM was examined. A flow cytometer was used to determine the accumulation of Rho123. In both PAC-resistant cell lines we observed a reduced Rho123 fluorescence compared to the sensitive cell line (Figure 2A).

The transport activity of the P-gp protein in PAC-resistant cell lines was also confirmed by the study of Calcein-AM accumulation using an inverted fluorescence microscope. During intravital imaging, the intracellular fluorescence of Calcein-AM was visible only in the sensitive cell line, but it was not observed in any of the resistant cell lines (Figure 2B).

### 2.4. MTT Analyses of Elacridar Effect on Resistance to Cytotoxic Drugs

**Figure 3:**
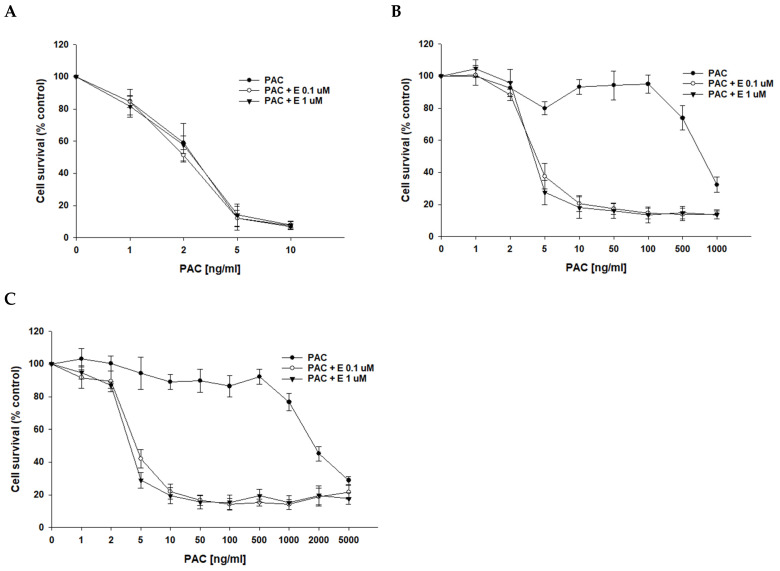
Elacridar (E) sensitizes PAC-resistant cell lines to PAC in vitro. PAC-sensitive cell line A2780 (**A**) and cell lines resistant to PAC, A2780PR1 (**B**) and A2780PR2 (**C**). The cells were seeded in 96-well plates. After 48 h, they were treated with PAC or PAC + E in concentrations of 0.1 or 1 μM. After 72 h of treatment, cell viability was determined using the MTT assay. Viability was expressed as a percentage of the untreated control (mean ± SEM).

After determining that P-gp protein is responsible for drug resistance in the investigated cell lines, we wanted to check if the inhibition of P-gp protein activity with elacridar, a selective P-gp inhibitor, at concentrations of 0.1 μM and 1 μM will increase cell sensitivity to cytotoxic drugs. As we used PAC-resistant cell lines in this study, the first cytostatic drug tested was PAC. In PAC-sensitive cell line A2780, we observed a very similar cell response to PAC treatment in the absence and presence of elacridar at both concentrations used (Figure 3A), resulting in very similar IC_50_ values (IC_50_ = 2.52 ng/mL vs. IC_50_ = 2.18 ng/mL vs. IC_50_ = 2.50 ng/mL) (Table 3). In contrast, in PAC-resistant cell lines, the simultaneous use of elacridar and PAC resulted in increased sensitivity to PAC, with a clear increase in sensitivity at concentrations of 5 ng/mL and at higher concentrations (Figure 3B,C). The comparison of PAC IC_50_ values in the absence and presence of elacridar at concentrations of 0.1 μM and 1 μM showed a strong reduction in IC_50_ values (Table 3). For both PAC-resistant cell lines, the treatment with 0.1 μM elacridar resulted in an increased sensitivity to PAC: 162-fold for A2780PR1 (IC_50_ = 4.66 ng/mL vs. IC_50_ = 755 ng/mL) and 397-fold for A2780PR2 (IC_50_ = 4.96 ng/mL vs. IC_50_ = 1970 ng/mL). The treatment with elacridar at a concentration of 1 μM resulted in a higher increase in sensitivity to PAC: 187-fold for A2780PR1 (IC_50_ = 4.04 ng/mL vs. IC_50_ = 755 ng/mL) and 483-fold for A2780PR2 (IC_50_ = 4.07 ng/mL vs. IC_50_ = 1970 ng/mL).

**Figure 4:**
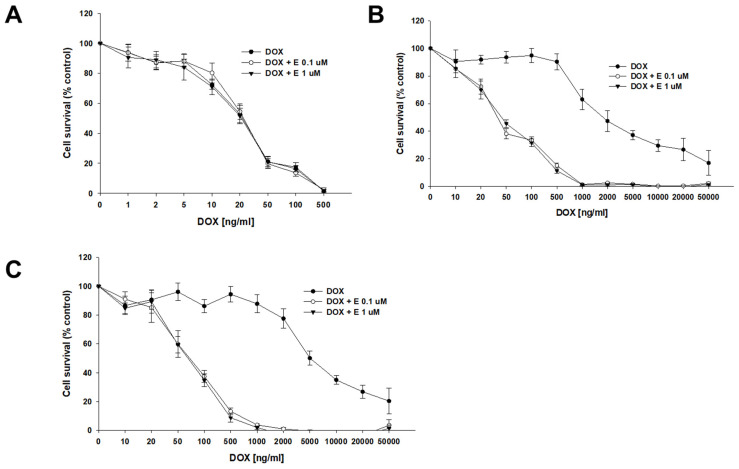
Elacridar (E) sensitizes PAC-resistant cell lines to DOX. PAC-sensitive cell line A2780 (**A**) and PAC-resistant cell lines A2780PR1 (**B**) and A2780PR2 (**C**) were seeded into 96-well plates. Cells were treated with DOX or DOX + E at concentrations of 0.1 or 1 μM. After 72 h of treatment, cell viability was determined using the MTT assay. Viability was expressed as a percentage of the untreated control (mean ± SEM).

DOX, another substrate of P-gp, was the second drug used in our study. The sensitivity of the A2780 cell line did not change after the use of elacridar at any concentration used (Figure 4A), resulting in very similar IC_50_ values (IC_50_ = 22.7 ng/mL vs. IC_50_ = 23.3 ng/mL vs. IC_50_ = 22.7 ng/mL) (Table 4). However, for PAC-resistant cell lines, we observed an increased sensitivity to DOX, starting from a concentration of 20 ng/mL in the A2780PR1 cell line and 50 ng/mL in the A2780PR2 cell line, respectively (Figure 4B,C). The DOX IC_50_ decreased by 46-fold in A2780PR1 (IC_50_ = 2033 ng/mL vs. IC_50_ = 44.4 ng/mL) and 92.8-fold in A2780PR2 (IC_50_ = 6292 ng/mL vs. IC_50_ = 67.8 ng/mL) in the presence of 0.1 μM elacridar. The use of elacridar in a 1 μM concentration caused a similar decrease in the DOX IC_50_ value. In the A2780PR1 cell line, we observed a 41-fold decrease in sensitivity (IC_50_ = 2033 ng/mL vs. IC_50_ = 50.0 ng/mL). In the A2780PR2 cell line, we observed a 101-fold decrease in sensitivity to DOX (IC_50_ = 6292 ng/mL vs. IC_50_ = 62.1 ng/mL).

**Figure 5:**
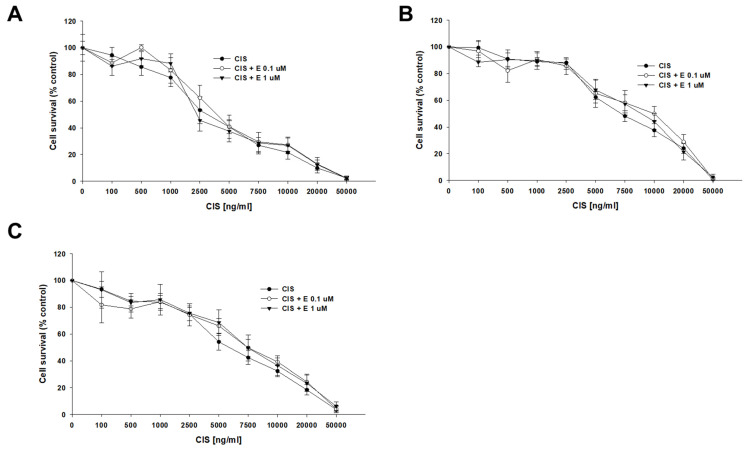
Elacridar (E) did not sensitize PAC-resistant cell lines to CIS. PAC-sensitive parental cell line A2780 (**A**) and PAC-resistant A2780PR1 (**B**) and A2780PR2 (**C**) were seeded into 96-well plates. The cells were treated with CIS or CIS + E at concentrations of 0.1 or 1 μM. After 72 h of treatment, cell viability was determined using the MTT assay. Viability was expressed as a percentage of the untreated control (mean ± SEM).

A co-treatment with CIS and elacridar did not change the sensitivity of any of the tested cell lines to CIS (Figure 5). The determined CIS IC_50_ values for the sensitive cell line were IC_50_ = 3763 ng/mL (CIS) vs. IC_50_ = 4567 ng/mL (CIS + 0.1 μM elacridar) and IC_50_ = 4050 ng/mL (CIS + 1 μM elacridar). Also, no statistical differences in CIS IC_50_ value were found for both PAC-resistant cell lines: for A2780PR1, IC_50_ = 7915 ng/mL (CIS) vs. IC_50_ = 9483 ng/mL (CIS + 0.1 μM elacridar) vs. IC_50_ = 9835 ng/mL (CIS + 1 μM elacridar) and for A2780PR2, IC_50_ = 6096 ng/mL (CIS) vs. IC_50_ = 7401 ng/mL (CIS + 0.1 μM elacridar) vs. IC_50_ = 7689 ng/mL (CIS + 1 μM elacridar) (Table 5).

### 2.5. Analysis of P-gp Activity in Elacridar-Treated Cell Lines

**Figure 6:**
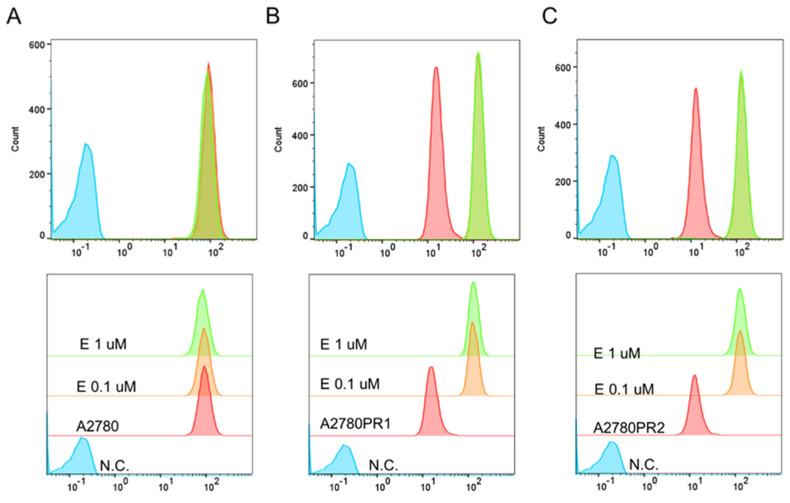
Flow cytometry analysis of intracellular accumulation of Rho123 in a drug-sensitive A2780 cell line (**A**) and PAC-resistant cell lines A2780PR1 (**B**) and A2780PR2 (**C**). The diagrams of fluorescence intensity show the fluorescence intensity in the untreated cell line (red) and cell lines treated with elacridar (E) at concentrations of 0.1 μM (orange) or 1 μM (green). N.C.—negative control without Rh123 (blue).

In the next step, we examined whether elacridar affects the transport activity of the P-gp protein in the tested cell lines. The analysis of the fluorescence of Rho123 accumulated in the A2780 cell line showed that the use of elacridar did not affect the dye removal from the cells (Figure 6A). However, in both PAC-resistant cell lines, elacridar treatment resulted in increased Rho123 accumulation, compared to untreated cells (Figure 6B,C). The accumulation occurred regardless of the concentration used.

**Figure 7:**
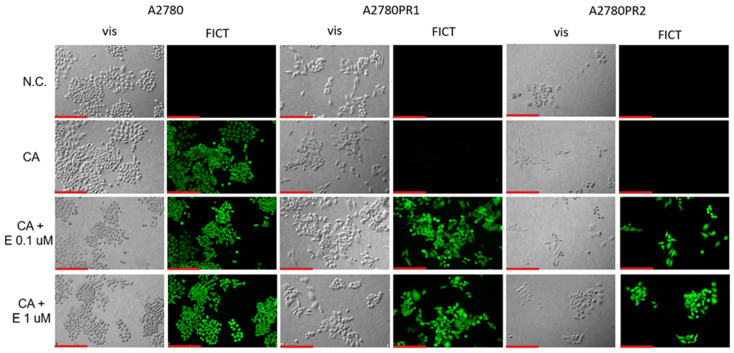
Fluorescence microscopy analysis of Calcein-AM accumulation (green) in the drug-sensitive A2780 cell line and PAC-resistant cell lines A2780PR1 and A2780PR2 in the absence or presence of elacridar (E) at concentrations of 0.1 μM and 1 μM. Objective 20×. Scale bar = 200 μm.

To confirm the effect of elacridar on P-gp activity, we investigated Calcein-AM accumulation in live cells, using inverted fluorescence microscopy techniques. We observed similar Calcein-AM fluorescence in PAC-sensitive A2780 cell lines in the presence and absence of elacridar in both concentrations used. However, in both PAC-resistant cell lines the Calcein-AM fluorescence was observed only after treatment with elacridar and the fluorescence intensity was similar with both elacridar concentrations used (Figure 7).

### 2.6. Expression and Activity of P-gp in Sensitive and PAC-Resistant Cancer Cell Lines in 3D Model

**Figure 8:**
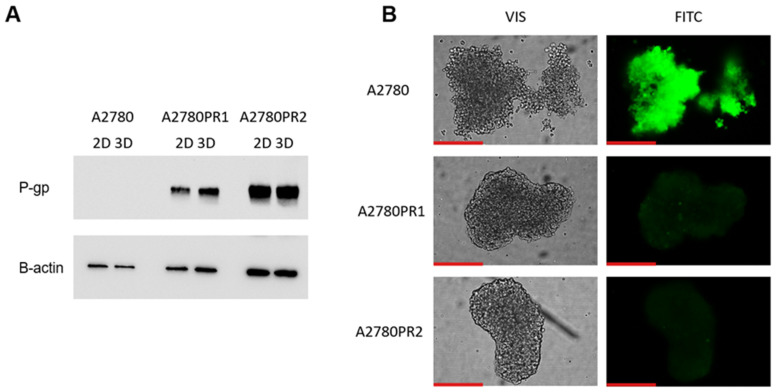
P-gp protein expression analysis in the A2780 and PAC-resistant cell lines growing as a monolayer (2D) or spheroids (3D). The cellular proteins were separated using 7% PAGE and transferred to a PVDF membrane, which was then immunoblotted with either primary Ab or HRP-conjugated secondary Ab. A primary anti-β Ab was used as a loading control for the cell lysates (**A**). Fluorescent microscopy analysis of Calcein-AM accumulation (green) in 3D spheroids derived from the drug-sensitive A2780 cell line and corresponding A2780PR1 and A2780PR2 PAC-resistant cell lines (**B**). Objective 20×. Scale bar = 200 μm.

The next part of our experiments was conducted to check if elacridar affects ovarian cancer cell sensitivity to cytotoxic drugs in a model that is more complex than a cellular monolayer. We chose a 3D spheroid model to achieve this goal. First, we compared the expression of P-gp protein in the 2D and 3D models, using the Western blot method (Figure 8A). In the sensitive cell line, both in the 2D and 3D models, we did not detect the P-gp protein expression. However, in PAC-resistant cell lines, both in 2D and 3D models, we observed a strong band corresponding to P-gp protein (Figure 8A). We also checked the transport activity of the P-gp protein in the 3D model. The spheroids were treated with Calcein-AM and then live spheroids were imaged with an inverted fluorescence microscope (Figure 8B). In the sensitive cell line, we observed a high fluorescence of dye. However, no Calcein-AM fluorescence was observed in any of the drug-resistant cell lines (Figure 8B).

### 2.7. Analysis of P-gp Activity in Elacridar-Treated Spheroids

**Figure 9:**
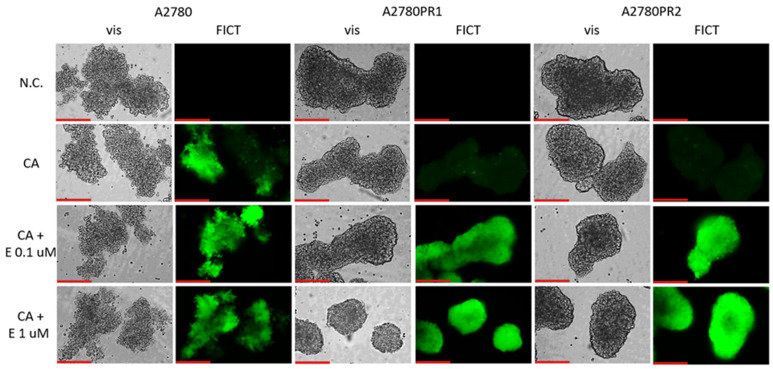
Fluorescence microscopy analysis of Calcein-AM accumulation (green) in spheroids derived from the drug-sensitive A2780 cell line and PAC-resistant cell lines A2780PR1 and A2780PR2 in the absence or presence of elacridar (E) at concentrations of 0.1 μM and 1 μM. Objective 20×. Scale bar = 200 μm.

We then tested whether the treatment of spheroids with elacridar would increase the accumulation of Calcein-AM (Figure 9). In the A2780 sensitive cell line, we observed similar fluorescence of the spheroids, regardless of elacridar presence. However, no Calcein-AM fluorescence was observed in any of the PAC-resistant cell lines in the absence of inhibitor. On the other hand, treatment with elacridar resulted in high Calcein-AM fluorescence in spheroids formed of PAC-resistant cell lines.

### 2.8. Analysis of the Effect of Elacridar on Response to Cytotoxic Drug Treatment in a 3D Model

**Figure 10:**
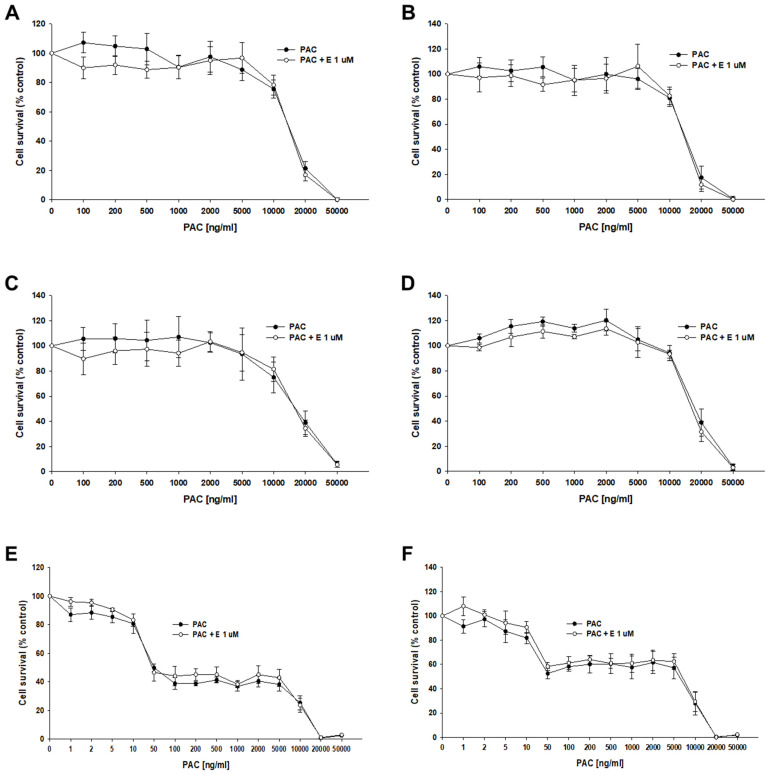
The effect of elacridar (E) on PAC resistance in PAC-resistant and PAC-sensitive cell lines cultured in 3D cell culture conditions. The spheroids were formed from 10 k (**A**,**C**,**E**) or 20 k (**B**,**D**,**F**) cells. A278 R1 cell line (**A**,**B**), A278 R2 cell line (**C**,**D**), and A2780 cell line (**E**,**F**). Then, they were treated with PAC or PAC + E at a concentration of 1 μM. After 72 h of treatment, cell viability was determined using the MTT assay. Viability was expressed as a percentage of the untreated control (mean ± SEM).

The next step of our research was to check the effect of elacridar at concentrations of 1 μM on the response to PAC in 3D spheroids. We used spheroids formed from 10,000 (10 k) and 20,000 (20 k) cells. After the formation of the spheroids, the cells were treated with PAC in the presence or absence of elacridar for 72 h. In both resistant and sensitive cell lines, we did not observe the effect of elacridar on spheroid response to PAC treatment in any amount of cells used (Figure 10A–F). In comparison, the IC_50_ value determined for treatment with PAC alone or co-treatment with elacridar did not show any significant differences (Table 6). The comparison of the IC_50_ values of the spheroids formed from 10,000 and 20,000 of cells in PAC-resistant cell lines did not show statistically significant differences in IC_50_ value as well (Figure 10A–D). In contrast, we observed a very high increase in IC_50_ value for PAC in spheroids formed from 10,000 and 20,000 cells of the A2780 cell line (IC_50_ = 56.7 ng/mL vs. IC_50_ = 5115 ng/mL), resulting in about a 90-fold increase in resistance (Figure 10 E,F), Table 6.

**Figure 11:**
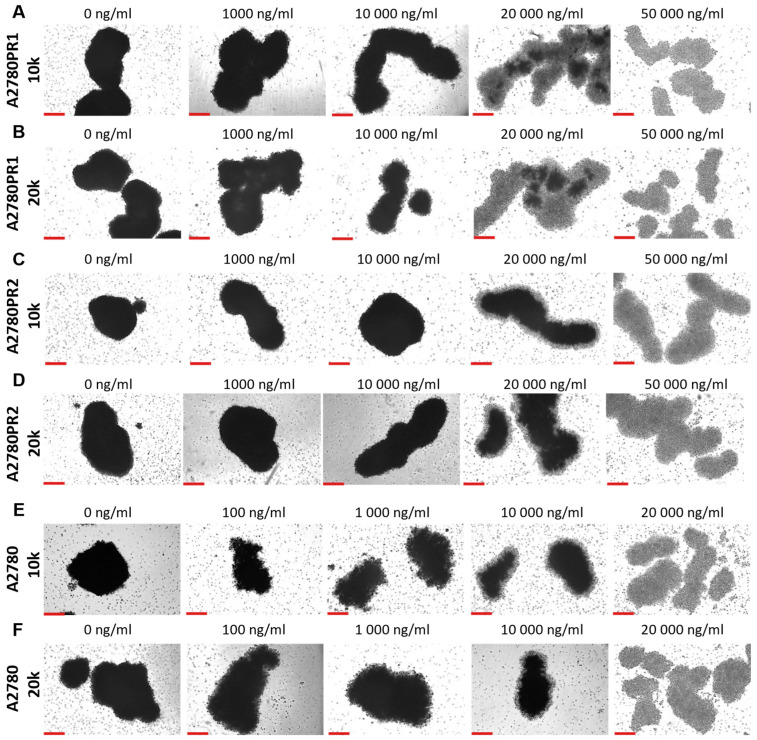
The viability visualization of spheroids formed from PAC-resistant, (**A**–**D**), and drug-sensitive, (**E**,**F**), cell lines after PAC treatment. We conducted the MTT assay in 3D conditions. Live cells convert tetrazolium salts to dark blue formazan, resulting in the spheroids’ dark color. A light color in spheroids means unviable cells. Objective 10×, scale bar = 200 μm.

During the MTT assay, we also examined the A2780, A2780PR1, and A2780PR2 cells under the microscope. In this experiment, the endoplasmic reticulum enzyme, particularly mitochondrial succinate dehydrogenase, catalyzed the cleavage of tetrazolium salts in living cells, leading to the formation of dark crystals [52]. We then took the pictures of the spheroids, in which the dark pigmentation represents viable cells and the lack of pigmentation represents unviable cells. In PAC-resistant cell lines, we observed a reduction in the number of darker cells in spheroids at a PAC concentration of 20,000 ng/mL. In the A2780PR1 cell line, we observed dark, live cells inside the spheroids and light, non-viable cells outside the spheroid. At the same PAC concentration, in A2780PR2, we observed some light, dead cells in the core of the spheroids; however, most of the cells showed a dark hue, corresponding to live cells. Completely dead, light spheroids are observed at a PAC concentration of 50,000 ng/mL in both PAC-resistant cell lines, as evidenced by the lack of precipitated formazan crystals (Figure 11A–D). However, in the sensitive cell line, the outer layer of spheroids started to be light at a concentration of 100 ng/mL, with a clear light core in spheroids at 10,000 ng/mL and the entire spheroid dead at 20,000 ng/mL of PAC (Figure 11E,F). The viability of spheroids was similar in spheroids formed from 10 k and 20 k cells.

**Figure 12:**
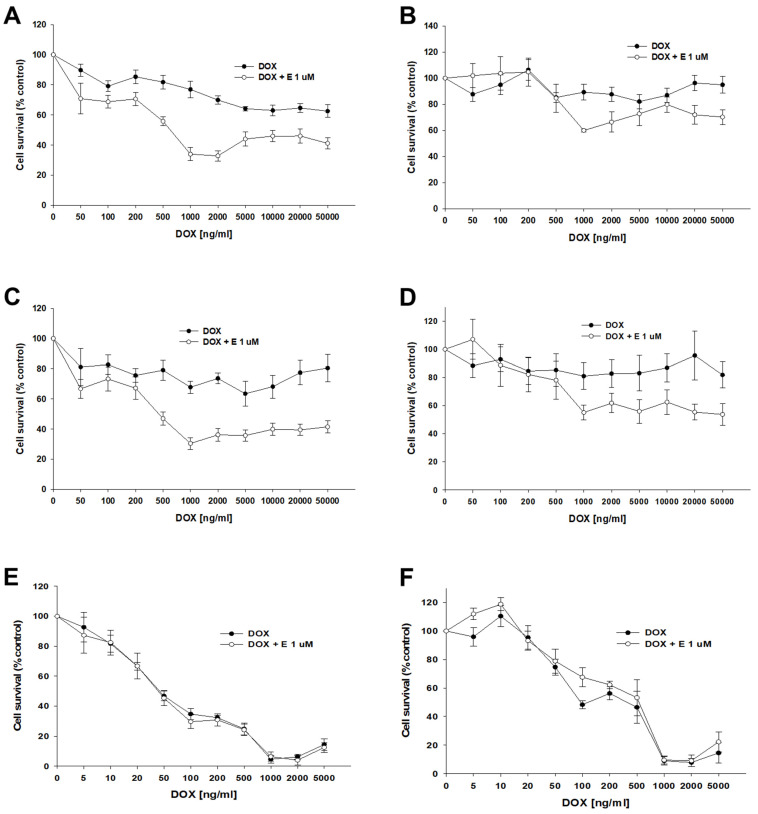
The effect of elacridar (E) on DOX resistance in PAC-resistant and PAC-sensitive cell lines cultured in 3D cell culture conditions. The spheroids were derived from 10 k, (**A**,**C**,**E**), or 20 k, (**B**,**D**,**F**), cells of the PAC-resistant cell lines A2780PR1, (**A**,**B**), and A2780PR2, (**C**,**D**), and the PAC-sensitive cell line A2780, (**E**,**F**). The cells were treated with DOX or DOX + E at a concentration of 1 μM. After 72 h of treatment, cell viability was determined using the MTT assay. Viability was expressed as a percentage of the untreated control (mean ± SEM).

Our next step was to test the effect of elacridar on response to another P-gp substrate, DOX, in spheroids. Spheroids formed from 10 k or 20 k cells were treated with DOX or DOX + elacridar at a concentration of 1 μM. After 72 h of incubation, the IC_50_ was determined by an MTT assay (Figure 12). In both PAC-resistant cell lines and both numbers of cells we were not able to determine the IC_50_ value for DOX (Table 7), (Figure 12A–D). The co-treatment with elacridar resulted in much higher sensitivity to DOX in spheroids formed from 10 k cells. There, we observed a higher sensitivity to DOX at every concentration used (Figure 12A,C) with a strong reduction in IC_50_ value (81.3-fold for A2780PR1 and 121-fold for A2780PR2) (Table 7—part A). On the other hand, in spheroids formed from 20 k cells, we did not observe significant changes in cell viability after DOX treatment, regardless of elacridar presence (Table 7—part B). In the A2780PR1 cell line, a very similar response curve was observed in the absence and in the presence of elacridar (Figure 12B). In the A2780PR2 cell line, at a DOX concentration of 1000 ng/mL or higher, we observed higher sensitivity to DOX in the presence of elacridar. However, the spheroid viability was always over 50% at every tested DOX concentration (Figure 12D).

For the sensitive cell line, we observed a very similar response curve towards DOX in the absence and presence of elacridar in spheroids formed from 10 k and 20 k cells (Figure 12E,F). However, we observe a strong difference between spheroids formed from 10 k and 20 k cells. Spheroids formed from 20 k cells were more resistant to DOX at concentrations from 5 to 500 ng/mL; this is reflected by a nearly 10-fold increase in IC_50_ value (48.7 ng/mL vs. 462 ng/mL).

**Figure 13:**
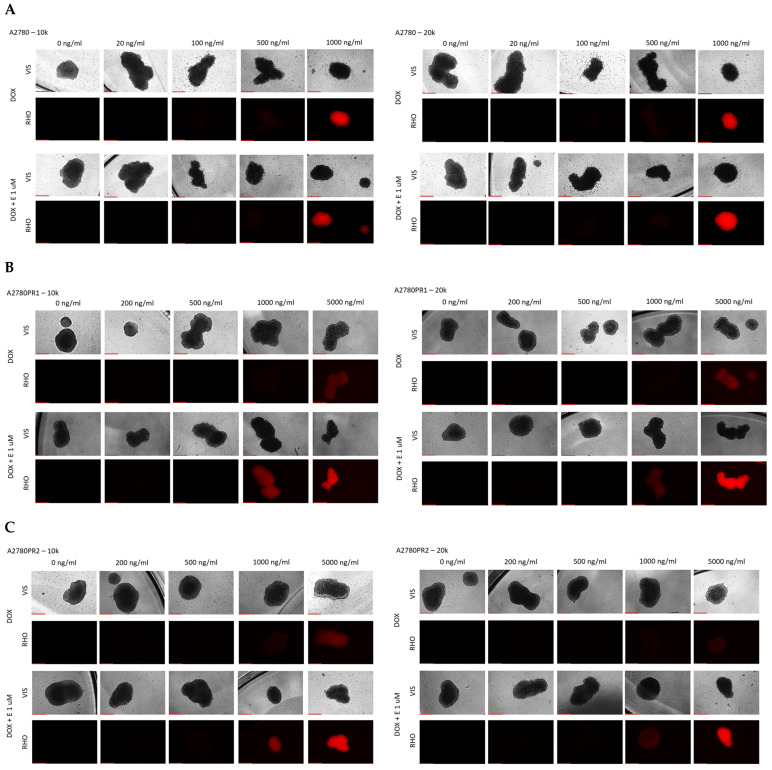
Fluorescence microscopy analysis of DOX accumulation (red) in spheroids derived from the drug-sensitive A2780 (**A**) cell line and PAC-resistant cell lines A2780PR1 (**B**) and A2780PR2 (**C**) in the absence or presence of elacridar (E) at a concentration of 1 μM. Objective 10×, scale bar = 250 μm.

Using the fluorescent property of DOX, we checked its accumulation in spheroids. For the A2780 cell line, we did not observe any change in DOX accumulation in the presence of elacridar. DOX fluorescence is visible at a concentration of 1000 ng/mL, regardless of elacridar presence and spheroid size (Figure 13A). In both PAC-resistant cell lines, we observed a low DOX fluorescence at a concentration of 5000 ng/mL. Co-treatment with 1 μM elacridar resulted in low DOX fluorescence at a concentration of 1000 ng/mL, and strong DOX fluorescence at a concentration of 5000 ng/mL in spheroids formed from 10 k and 20 k cells in both of the resistant cell lines (Figure 13B,C).

## 3. Discussion

Drug resistance in cancer is the main reason for the low efficiency of conventional chemotherapy. Some cancers are primarily resistant to chemotherapy, while others, like ovarian cancer, develop drug resistance during treatment [53]. At a cellular level, drug resistance is very often associated with an overexpression of drug transporters from the ABC family. During previous decades, scientists investigated different strategies to increase the efficiency of chemotherapy. One approach is the inhibition of drug transporters’ function. In recent years, many new specific inhibitors of ABC transporters, third generation inhibitors, have been developed [54], and among them, elacridar [33].

For this study, we selected a model of drug resistance development in ovarian cancer composed of the drug-sensitive cell line A2780 and its two sublines resistant to PAC, A2780PR1 and A2780PR2 [55], to study the effect of elacridar.

In both resistant cell lines, we observed extremely high resistance to PAC and a very high level of resistance to DOX. In contrast, resistance to CIS increased only slightly and only in the A2780PR1 cell line.

As the P-gp is often associated with PAC and DOX resistance [56] we analyzed the expression of *MDR1*/P-gp in investigated cell lines. The analysis of mRNA expression and protein levels in A2780PR1 and A2780PR2 cell lines shows a lack of expression in the drug-sensitive cell line and a very high expression level in drug-resistant cell lines. Immunofluorescence analysis indicated that P-gp is localized mainly in the cell membrane.

As P-gp is a drug efflux pump, we also assessed if its expression level is associated with its activity in our model. The flow cytometry analyses, conducted using Rhodamine123, a known substrate of P-gp [57], confirmed heightened P-gp transport activity in the cells exhibiting P-gp overexpression and PAC resistance.

To further confirm the transporter activity of P-gp we also conducted live analysis using inverted fluorescence microscopy and Calcein-AM as a P-gp substrate. The observation of substrate accumulation in live cells indicates that calcein is not present within drug-resistant cells, implying that P-gp actively disposed of Calcein-AM or its fluorescent metabolite.

All of these results confirm that P-gp is an important factor in the drug-resistant phenotype of these cells—a trait commonly observed in PAC-resistant cancer cells [58].

In summary, this “preliminary research” confirmed that very high PAC and DOX resistance in the investigated cell lines is associated with P-gp overexpression and activity. Thus, we chose this model to study the effect of elacridar on drug resistance.

In our MTT assays, we observed that the addition of elacridar in concentrations of 0.1 μM and 1 μM results in the re-sensitization of PAC-resistant cell lines to PAC and DOX, a substrate of P-gp, but caused no change in resistance to CIS, which is not a P-gp substrate [53]. We also did not observe any effect of elacridar treatment on the drug-sensitive cell line. These results suggest that the effect of elacridar is P-gp dependent.

Our flow cytometry analysis indicated that elacridar inhibits P-gp activity in PAC-resistant cell lines and this was further confirmed by a Calcein-AM accumulation study.

In our research, we used elacridar in two concentrations: 0.1 μM and 1 μM. Both concentrations show a strong inhibitory effect on P-gp activity, reflected in a strong increase in sensitivity to PAC and DOX. However, using different molecular and cellular biology techniques, we did not observe significant differences between the concentrations, which suggests that the concentration of 0.1 μM is sufficient to inhibit P-gp activity in our model.

A similar range of elacridar concentration was used in other cell line studies. In experiments conducted on the daunorubicin-resistant human promyelocytic leukemia cell line HL60/DNR, a treatment with 0.1 μM elacridar resulted in re-sensitization to daunorubicin (40-fold decrease in IC_50_) and mitoxantrone (57-fold decrease in IC_50_) [34]. In the chronic myeloid leukemia (CML) cancer cell lines K562 and LAMA-84 and imatinib-resistant cell cultures, elacridar at a concentration of 0.25 μM was tested in combination with imatinib; the flow cytometry analysis confirmed the successful inhibition of ABC transporter activity, leading to a reversal of imatinib resistance. The increase in the sensitivity (the decrease in IC_50_) was 5-fold and 10-fold, depending on the tested cell line [59]. In non-small cell lung (NSCL) cancer cell lines, elacridar at a concentration of 0.25 μM, combined with docetaxel, increased the sensitivity of docetaxel-resistant cell lines from 28.9-fold to over 3333.3-fold, depending on the cell line treated with the inhibitor [60]. In CIS-resistant IGROVCDDP ovarian cancer cell lines with P-gp overexpression, treatment with 0.25 μM elacridar caused increased cell sensitivity to several P-gp substrates (docetaxel, PAC, epirubicin, and vinblastine), though it has not affected resistance to drugs that are not carried by this protein (SN-38, 5-FU, and methotrexate) [37]. In the docetaxel-resistant human prostate cancer cell lines DU-145R and 22RV1, exhibiting positive P-gp expression, the addition of 0.25 μM elacridar enabled the reversal of docetaxel resistance. In experiments conducted on the renal carcinoma cell line 786-O, elacridar was used at concentrations of 2.5 μM and 5 μM in combination with sunitib, resulting in an enhanced cytotoxic effect through the inhibition of P-gp activity [61].

In summary, in most of the cell culture studies, elacridar at a concentration below 1 μM effectively inhibited P-gp activity, leading to varying decreases in drug resistance. In our model, we observed about a two-fold higher expression of *MDR1*/P-gp in the A2780PR2 cell line compared to the A2780PR1 cell line. Treatment with elacridar resulted in about a two-fold greater decrease in resistance in the A2780PR2 cell line, compared to the A2780PR1 cell line. Thus, we concluded that the effect of this inhibitor is proportional to P-gp expression level—stronger expression equals stronger effect.

All of these results confirm that elacridar inhibits P-gp activity and sensitizes drug-resistant cancer cells to cytotoxic drugs that are P-gp substrates, suggesting that elacridar can be a good candidate for an anticancer drug to increase chemotherapy effectiveness. The experiments described above were conducted in a 2D cell culture, which is the best model to study cellular mechanisms of drug resistance, but does not simulate the more complicated conditions of the high cell density and extracellular matrix environment present within tumors [62,63]. To better characterize the response to elacridar we carried out a series of similar experiments on a cell culture grown as 3D spheroids. This model was well characterized and described by us previously [48].

The initial assessment of P-gp protein expression confirms that P-gp expression is heightened in drug-resistant cell cultures grown both in 2D and 3D conditions and there is no significant change in P-gp quantity in samples derived from 2D and 3D cell lysates. This observation is consistent with our previous study where we observed a similar level of *MDR1* gene expression in 2D and 3D cell culture models [47,48]. Thus, the overexpression of P-gp seems to be a feature of drug-resistant cells growing both as a 2D monolayer or 3D spheroids.

The fluorescent microscopy analysis of spheres treated with Calcein-AM points out that the substrate has been removed from the spheres grown from PAC-resistant cell lines, indicating high activity of P-gp in these aggregates. In contrast, high fluorescence has been observed in spheroids formed from the A2780 cell line, suggesting that Calcein-AM easily diffuses into the spheroid structure.

The analysis of Calcein-AM accumulation following the addition of elacridar to spheroids showed that elacridar is capable of penetration through cellular aggregates and successful P-gp inhibition in 3D structures. This indicates that elacridar can be considered as a potent drug increasing chemotherapy effectiveness. In the next step of our study, we wanted to verify this hypothesis.

To compare if the effect of drugs is spheroid size dependent, spheroids were formed from 10 K and 20 K cells per well. In the case of drug-resistant cell lines, the inhibitor had no significant effect on the overall cell response to PAC in PAC-resistant cell lines and the IC_50_ values were very similar in spheroids formed from 10 K and 20 K cells. This result suggests that other mechanisms are also related to PAC resistance in 3D spheroids. In contrast to drug-resistant cell lines, the IC_50_ value for PAC was drastically different in the drug-sensitive cell line and it increased about 90-fold in spheroids formed from 20 K cells. In the case of drug-resistant cell lines, in both 10 K and 20 K cells, and in the presence and absence of elacridar, we observe a two-step response curve that is flat in the lower PAC concentration, and only very high PAC concentrations are able to kill cells in spheroids. In contrast, in the A2780 cell line we observed a four-step response curve. Spheroids start to gain sensitivity at concentrations over 10 ng/mL. At the concentration of 50 ng/mL, we observe viability at the level of about 45% of control in spheroids formed from 10 K cells and at the level of over 50% in the case of cells formed from 20 K cells. Further increases in PAC concentration up to 5000 ng/mL (100-fold) did not change the viability of the spheroids and only very high concentrations of PAC were able to kill all cells in the spheroids.

The differences between spheroids formed from drug-sensitive and drug-resistant cell lines do not result from the presence of P-gp in drug-resistant cell lines, as the blocking of P-gp activity by elacridar did not change the response curve to PAC. The explanation of this phenomenon probably lies in the structure of the spheroids and/or the expression of other drug-resistant genes/proteins. A typical spheroid is composed of several different zones: the proliferation zone formed by the outer layers of cells, the quiescent cell zone present inside, and in the case of bigger spheroids, there also is the necrotic zone that is present in the very center [64]. As PAC is an antimitotic drug, it targets mainly proliferating cells in the outer zone of spheroids formed from A2780 cells, while cells in the inner, quiescent zone are not sensitive to PAC in lower concentrations and only a very high PAC concentration is able to kill quiescent cells, probably following another mechanism. The quiescent zone is likely bigger in the spheroids formed from 20 K of cells, resulting in the increase in the IC_50_ value, although the response curve is nearly identical. This prompted the question of why we do not observe such a response curve in the case of drug-resistant cells treated with elacridar. Looking at Figure 8 and Figure 9 we can notice that spheroids formed by drug-resistant cells are high-density spheroids, while spheroids formed by A2780 cells seem to form medium-density spheroids. As PAC is a very big molecule, exhibiting low diffusion properties in cancer [65], a high density of cells can very effectively protect cells inside the spheroids. Previously, we observed a very high PAC resistance level in dense spheroids formed from the drug-sensitive W1 ovarian cancer cell line. The high increase in PAC resistance between 2D monolayer and 3D spheroid conditions was also observed by others [66]. In a breast cancer study, the resistance of spheroids to PAC was dependent on spheroid density [67]. A similar observation was made in 3D spheroids formed from MDA-MB-231, a breast cancer cell line [68]. All of these results suggest that an increase in PAC resistance can be associated with the limited potential of PAC to diffuse into the dense cellular structure.

Next, we investigated the impact of elacridar on response to DOX treatment in spheroids. In the A2780 cell line, we observed a typical, concentration-dependent linear response, which suggests that DOX easily diffuses through the spheroid structure. An increase in IC_50_ value in spheroids formed from 20 K cells in comparison to spheroids formed from 10 K cells is probably a consequence of bigger spheroid size.

Different response curves were observed in PAC-resistant cell lines. In general, the DOX response curve was nearly flat, although in spheroids formed from 10 K cells, some effect was observed in the A2780PR1 cell line. Elacridar treatment resulted in an increased sensitivity to DOX, especially in spheroids formed from 10 K cells. Also, elacridar treatment resulted in the accumulation of DOX in spheroids at the concentration of 1000 ng/mL vs. 5000 ng/mL without elacridar, and this effect was more visible in spheroids formed from 10 K cells.

Thus, results from both the MTT assay and DOX accumulation assay suggest that elacridar indeed increases the sensitivity to DOX in the mechanism that is dependent on P-gp-inhibition. However, even in the presence of elacridar, DOX is not able to kill all of the cells in spheroids, although it is present in every zone of the spheroids, probably because DOX mainly targets proliferating cells, so it kills cells in the proliferation zone only [69]. Again, we observed difference between spheroids grown from A2780 cells and spheroids formed from the A2780PR1 and A2780PR2 cell lines. Even in the presence of elacridar, DOX was not able to kill all of the cells in drug-resistant cell lines, while in drug-sensitive cell line it could do so. We suppose that this difference is a consequence of the different cell densities of the discussed spheroids. A similar observation was made by Immatura et al. in spheroids formed from different breast cancer cell lines. MCF-7, HCC-1954, and MDA-MB-231 cell lines formed loose spheroids, and compared with the 2D model, revealed similar sensitivities to DOX. In contrast, BT-549, BT-474, and T-47D formed high density spheroids and revealed high resistance to DOX, compared with the 2D model [67].

One more factor can also be involved in the higher resistance of spheroids from drug-resistant cell lines to PAC and DOX. The increase in drug resistance can be associated with a higher expression of ECM molecules and other drug-resistant genes in both PAC-resistant cell lines. It is well established that a high expression of ECM components in tumors limits the diffusion of therapeutic agents [63,70], and causes some cytostatic agents like PAC and DOX to bind to cellular macromolecules, limiting their availability to the cancer cells [63].

The interaction of cancer cells with ECM components can also result in drug resistance through a mechanism designated as cell adhesion-mediated drug resistance (CAM-DR) [71,72]. This leads to the activation of intracellular signaling pathways, the expression of pro-survival genes and, in consequence, a higher resistance to drug-induced apoptosis [71,72].

Previously, we observed the expression of many ECM-related genes in the A2780PR1 cell line [48,73,74,75]. In both resistant cell lines, we also observed an increased/decreased expression of many other genes associated with drug resistance [76]. Some of these genes, like *SAMD4* or *S100A3,* were upregulated in PAC-resistant cell lines [48], while others, like *SEMA3A* and *PTPRK,* were downregulated [76,77]. PTPRK is an intracellular phosphatase and the downregulation of its expression is associated with an increased activation of intracellular signaling pathways, which results in higher drug resistance [77]. *SEMA3A* is a suppressor gene, and the loss of its expression is associated with cancer progression and increased drug resistance [78]. Also, the increased expression of *S100A3* was observed in the progression of different cancers [78]. This indicates that tissue-specific drug resistance mechanisms and mechanisms leading to signaling pathway activation can effectively limit chemotherapy effectiveness even in the case of drug transporter inhibition.

In conclusion, we carried out a study concerning the usefulness of elacridar, a third generation P-gp inhibitor, in increasing the effectiveness of chemotherapy. Our 2D study fully confirmed the results obtained by other researchers, supporting that elacridar very effectively inhibits P-gp activity, leading to increased drug sensitivity.

Our results also revealed that elacridar effectively inhibits P-gp activity in 3D spheroids. However, a mechanism of resistance in 3D spheroids is much more complicated, as factors like drug diffusion properties, the density of the cells, the expression of ECM molecules, and other drug-resistant genes are also involved.

We believe that further research with elacridar will result in increased chemotherapy effectiveness, at least in lower density tumors and leukemias.

## 4. Materials and Methods

### 4.1. Reagents and Antibodies

Paclitaxel (PAC), cisplatin (CIS), doxorubicin (DOX), and elacridar were purchased from Selleckchem, (Houston, TX, USA). MEM medium, fetal bovine serum (FBS), penicillin, streptomycin, amphotericin B (25 μg/mL), trypsin EDTA solution, and L-glutamine were acquired from Sigma (St. Louis, MO, USA). DPBS was purchased from Corning (Corning, NY, USA). Cell Proliferation Kit I (MTT)—Thiazolyl Blue Tetrazolium Bromide, Calcein-AM (206700-1MG), and Rhodamine 123 (Rho 123) were also purchased from Sigma (St. Louis, MO, USA). Mouse monoclonal anti-P-glycoprotein antibody (C219) (Alx-801-002-c100) (P-gp) was obtained from Enzo, Farmingdale, NY, USA. Anti-GAPDH antibody, anti-β-actin antibody, and goat anti-mice horseradish peroxidase (HRP)-conjugated Ab were purchased from Proteintech, Rosemont, IL, USA (SA00001-1-A). Fluorescent Alexa Fluor^®^488 AffiniPure™ Donkey anti-mouse IgG (H + L) (715-545-150, 1:200) was obtained from Jackson ImmunoResearch Laboratories (Jackson ImmunoResearch Laboratories, Cambridgeshire, UK). M-MLV reverse transcriptase kit (28025013) and RnaseOUT (Invitrogen by ThermoFisher, Waltham, MA, USA, 10777019) were acquired from Invitrogen by ThermoFisher, US. TakyonTM ROX SYBR^®^ MasterMix blue dTTP was obtained from Eurogentec (Kaneka Eurogentec, Liège, Belgium). All Western blot reagents (membranes, gels, and protein marker) were purchased from Bio-Rad (Bio-Rad Laboratories Ltd., Watford, Hertfirdshire, UK).

### 4.2. Cell Culture

The A2780 human ovarian cancer cell line was purchased from ATCC (Manassas, VA, USA). The paclitaxel-resistant sublines (A2780PR1 and A2780PR2) were obtained by treating A2780 cells with gradually increased concentrations of the drug, with 300 ng/mL being the final concentration of paclitaxel for A2780PR1 and 1100 ng/mL being the final concentration of paclitaxel for A2780PR2. The cells were cultured as a monolayer in an MEM medium containing 10% (*v*/*v*) fetal bovine serum, 2 pM L-glutamine, streptomycin (100 U/mL), penicillin (100 U/mL), and amphotericin B (25 μg/mL) in a 5% CO_2_ atmosphere at 37 °C.

### 4.3. RNA Isolation, cDNA Synthesis, and QPCR

RNA isolation from all cell lines was performed using the Gene Matrix Universal RNA Purification Kit (EURx, Ltd., Gdańsk, Poland) according to the manufacturer’s instructions. The amount of isolated RNA was then determined by measuring absorbance (260 and 280 nm).

A total of 1.5 μg of RNA was used for reverse transcription to synthesize cDNA. cDNA was obtained using the CFX Opus 96 Real-Time PCR System (Bio-Rad, Hercules, CA, USA). Each single sample consisted of 1.5 μg RNA, 1 μL oligodT18A (IBB PAN, Warsaw, Poland), and 1 μL dNTP Mix (ThermoFisher, Waltham, MA, USA, R0192) and was denatured at 65 °C for 5 min. Then, 4 μL 5X First Strand Buffer, 2 μL DTT, 0.5 μL RnaseOUT, and 0.5 μL M-mLV RT were added to the mixture and the reaction was performed for 60 min at 37 °C and 15 min at 75 °C.

Real-time PCR was performed using the CFX Opus 96 Real-Time PCR System (Bio-Rad, USA). The reaction mixture for one sample was prepared by mixing 12.5 μL of TakyonTM ROX SYBR^®^ MasterMix blue dTTP (Eurogentec, Liège, Belgium), 1 μL of each sequence-specific primer (7.5 μM) from Oligo.pl (Warsaw, Poland) (Table 8), 9.5 μL of nuclease-free water, and 1 μL of cDNA solution. As a negative control, nuclease-free water instead of cDNA was used.

The real-time PCR reaction was carried out under the following conditions: 95 °C for 15 min (initial denaturation), 45 cycles; 95 °C for 15 s (denaturation); 60 °C for 30 s (primer annealing); 72 °C for 30 s (extension); and 72 °C for 30 s (final extension). Glyceraldehyde-3-phosphate dehydrogenase (GADPH) was used as an internal reference control (housekeeping gene).

Results were analyzed using the CFX Maestro (Bio-Rad v2.3 (5.3.022.1030) software). Gene expression analysis was performed using the relative quantification (RQ) method.

Gene expression analysis was performed using the relative quantification (RQ) method, which evaluates differences in gene expression relative to a calibrator (the RQ of the calibrator = 1). Drug-sensitive cell line A2780 served as a calibrator in the study. The RQ values were calculated using the standard formula: RQ = [sample (drug-resistant line) − calibrator (drug-sensitive line)]. Graphical representations of the results were generated using SigmaPlot software, version 15.0 (Systat Software, Inc., San Jose, CA, USA)

### 4.4. Two-Dimensional MTT Assay

Ovarian cancer cells were seeded in a 96-well plate in a quantity of 3000 cells per well in 200 μL of cell culture medium. After 48 h, the medium was replaced and the cells were treated with elacridar (0 μM, 0.1 μM, and 1 μM) and appropriate concentrations of drugs (PAC, DOX, or CIS), and then the cells were cultured for another 72 h. Next, the MTT test was performed. In each well, the medium was replaced with 170 μL of media mixed with 100 μg of MTT reagent and a 1 h incubation followed. Next, the medium was removed, and the obtained formazan crystals were dissolved in 200 μL of DMSO. After that, the absorbance was measured at wavelengths of 570 nm and 720 nm using Synergy LX Multi-Mode Reader by BioTek Instruments, Inc. (Winooski, VT, USA). From the results obtained, the IC_50_ was determined.

### 4.5. Three-Dimensional MTT Assay

Ovarian cancer cell lines were plated into nonadherent surface 96-well plates (BRAND plates inter Grade, F-bottom, 781,902 (Merck, Rahway, NJ, USA)) at 10,000 (10 k) or 20,000 (20 k) cells per well (50,000 or 100,000 cells/mL). After 48 h of culture, 100 μL of medium was replaced with 100 μL of medium with the elacridar (0 μM, 0.1 μM, or 1 μM) and appropriate concentrations of PAC or DOX, respectively. After 72 h of incubation of the spheroids with drugs and the inhibitor, the MTT test was performed with Cell Proliferation Kit I (Roche 11465007001). First, 100 μL of medium was removed from the wells, and 10 μL of MTT labeling reagent was added; the plates were then placed in the incubator for 4 h. Some cell lines grown in 3D conditions were photographed using the inverted microscope Leica DMi8 (Leica, Wetzlar, Germany). Next, 100 μL of solubilization buffer was added and the spheroids were incubated overnight in the culture incubator. The next day, photos of spheroids were taken for lines A2780 and PAC-resistant cell lines treated with the appropriate concentration of PAC. Next, absorbance readings were taken at 570 nm and 720 nm using Synergy LX Multi-Mode Reader by BioTek Instruments, Inc. (Winooski, VT, USA). From the results obtained, the IC_50_ was determined.

### 4.6. Immunofluorescence

The cells were cultured on coverslips in 24-well plates in a quantity of 10,000 cells per well. When the cells reached 70% confluence, the culture medium was removed and cells were rinsed with phosphate-buffered saline (PBS). Next, the cells were fixed and permeabilized by 15-min incubation with an ice-cold acetone/methanol (1:1) solution at room temperature (RT). After washing with PBS, the cells were blocked with 3% BSA solution for 30 min in RT and then incubated with the primary antibody, anti-P-gp (C219, 1:50) for 2 h at RT. The cells were then washed three times with PBS and incubated with a secondary antibody (Alexa Fluor^®^488-conjugated Donkey anti-mouse) for 1 h at RT. Next, the coverslips were washed three times with PBS, and adhered to the microscope slides with a DAPI-containing mounting medium (Sigma, St. Louis, MO, USA F6057), allowing the visualization of the nuclei. The expression of P-gp protein in the investigated cell lines was analyzed using a fluorescence microscope (Zeiss Axio-Imager.Z1, Oberkochen, Germany) and Zen Blue v3.3 software.

### 4.7. Live-Cell Fluorescence (CA Accumulation) (2D)

The cells were seeded into 24 well plates (1 × 10^4^ cells per well). After 72 h of growth, the cells were treated with elacridar at a concentration of 0.1 or 1 μM and incubated for 1 h. Next, the cells were again treated with the same concentration of elacridar with Calcein-AM in a final concentration of 0.25 μM. Following another 1-h incubation, the cells were washed with a cold solution of 50 μM verapamil in PBS. The washing was repeated three times and microscope observation followed. The pictures were taken with the inverted microscope Leica DMi8 (Leica, Wetzlar, Germany) at 20× magnification, for FITC and VIS channels.

### 4.8. Live-Cell Fluorescence (CA Accumulation) (3D)

The cells were seeded into non-adherent 96-well plates (5 × 10^4^ cells wells per well). After 5 days, the cells were treated with elacridar at a concentration of 0.1 μM or 1 μM, and incubated for 2 h. Next, cells were again treated with a 0.1 μM or 1 µM concentration of elacridar with 0.25 µM of Calcein-AM added to every well except the negative control. Following another 1 h incubation, the spheroids were washed with a cold solution of 50 μM verapamil in PBS. The washing was repeated three times and microscope observation followed. The pictures were taken with the inverted microscope Leica DMi8 (Leica, Wetzlar, Germany) at 40× magnification, for FITC and VIS channels.

### 4.9. Protein Isolation and Western Blot Analysis

Cells (1 × 10^6^ cells/300 μL of lysis buffer) were washed 3 times with PBS containing Ca/Mg ions. Then, the cells were lysed using RIPA buffer containing a protease inhibitor cocktail (Roche Diagnostics GmbH, Mannheim, Germany) for 80 min on ice. The lysates were centrifuged at 13.4 × 10^3^ rpm for 30 min at 4 °C, and protein concentrations were determined using the Bradford protein assay system (Bio-Rad Laboratories, Hemel Hempstead, UK). The wells were loaded with 15 μg of the protein resuspended in 4X loading buffer (Bio-Rad Laboratories, Hemel Hempstead, UK) with 10% β-mercaptoethanol. After separation on a 4–20% mini-PROTEAN^®^ TGX™ precast gel, performed with the SDS-PAGE technique, the proteins were transferred to a nitrocellulose membrane using the Trans-Blot^®^™ transfer system (Bio-Rad Laboratories, Hemel Hempstead, Hertfordshire, UK), and then blocked with 5% milk in TBS/Tween (0.1 M Tris-HCl, 0.15 M NaCl, 0.1% Tween 20) and incubated overnight in primary antibodies against P-gp (Enzo, Alx-801-002-c100) at a 1:400 dilution. Next, the 3-h long incubation with mice HPR-labeled secondary antibodies (Proteintech, Rosemont, IL, USA, SA00001-1-A, dilution:1:10,000) followed. Signals were developed using a chemiluminescence detection system (ECL, Femto Super Signal Reagent, Waltham, MA, USA) and ChemiDoc™ (Bio-Rad Laboratories, Hemel Hempstead, UK). The protein loading was normalized by reblotting the membranes with mice anti-actin Ab (Proteintech, 66009-1-Ig), at a 1:7500 dilution and goat anti-mice HRP-conjugated Ab (Proteintech; SA00001-1-A) at a 1:10,000 dilution).

### 4.10. Flow Cytometry Analysis

To determine P-gp efflux activity in drug-sensitive and drug-resistant cell lines and to determine the impact of elacridar, Rhodamine 123 (Rho123) fluorescence was measured. The cell suspensions (1 × 10^6^ cells/mL in cell culture medium) were treated with elacridar at a concentration of 0.1 or 1 μM and incubated for 1 h at 37 °C with an agitation of 800 rpm. Next, Rho123 in a final concentration of 1 μg/mL was added and the cells were incubated in these conditions for another hour. Then, the cells were placed on ice, centrifuged at 200× *g* for 5 min in 4 °C, washed twice in ice-cold PBS with 50 μM verapamil and immediately analyzed using a MACSQuant 10 cytometer (Miltenyi Biotec, Bergisch Gladbach, Germany)with the FACS-FlowJo 10.9 software program. In every analysis, 10,000 events were recorded. The fluorescent emission was measured at 488 nm.

### 4.11. Analysis of DOX Accumulation in Spheroids

Ovarian cancer cell lines were plated into nonadherent surface 96-well plates (BRAND plates inter Grade, F-bottom, 781,902 (Merck)) in quantities of 10,000 (10 k) or 20,000 (20 k) cells per well (50,000 or 100,000 cells/mL). Cell culture was continued for two days until stable spheroids formed. Then, the spheroids were treated for 72 h with the appropriate concentration of DOX (resistant lines: 0 ng/mL, 200 ng/mL, 500 ng/mL, 1000 ng/mL, and 5000 ng/mL; sensitive cell line: 0 ng/mL, 20 ng/mL, 100 ng/mL, 500 ng/mL, and 1000 ng/mL) and 1 μM elacridar. Then, the pictures were taken at a wavelength of 650 nm with a Leica DMi8 inverted fluorescence microscope (Leica, Wetzlar, Germany), using a 10× objective.

## Figures and Tables

**Table 1:** A list of P-gp inhibitors (based on [11,15]).

I-Generation	II-Generation	III-Generation
cyclosporin A, quinidine, quinine, reserpine, tamoxifen, tomoxifene, toremifene, verapamil, yohimbine	biricodar citrate (VX710), dexniguldipine, dexverapamil, dofequidarfumerate, S9788, valspodar (PSC 833)	annamycin, biricodar, CDP41251, elacridar (F12091, GG918), HM30181, laniquidar (R101933), mitotane (NSC-38721), ONT-093, R10933, tariquidar (XR9576), zosuquidar (LY335979)

**Table 2:** The summary of cell line resistance to PAC, DOX, and CIS treatment in the 2D cell culture condition. The PAC, DOX, and CIS IC_50_ values are indicated for each cell line. In every case, the drug resistance in the parental A2780 cell line was assigned a value of one. Underlined values indicate multiplicities of resistance when compared to the A2780 cell line. The up arrows indicate an increase in IC_50_ compared to the A2780 cell line. * *p* < 0.05 and ** *p* < 0.01.

Cell Line	PAC IC_50_ (ng/mL)	DOX IC_50_ (ng/mL)	CIS IC_50_ (ng/mL)
A2780	2.52	22.7	3763
	(1.69–3.78)	(17.2–33.3)	(1785–9117)
	1	1	1
A2780PR1	755	2033	7915
	(653–946)	(886–4328)	(4784–14,901)
	307 ↑ **	78.0 ↑ *	2.10 ↑ *
A2780PR2	1970	6292	6096
	(1390–3016)	(2631–9420)	(4013–8889)
	781 ↑ **	278 ↑ **	1.62 ↑

**Table 3:** The summary of cell line resistance to PAC treatment in the 2D cell culture condition in the absence and presence of elacridar at concentrations of 0.1 and 1 μM. The PAC IC_50_ values are indicated for each cell line. The PAC resistance in the cell lines treated with PAC alone was assigned a value of one for every cell line. Underlined values indicate multiplicities of sensitivity in the presence of elacridar with respect to cells treated with PAC alone. The down arrows indicate a decrease in IC_50_ value compared to control. ** *p* < 0.01.

Cell Line	Control PAC IC_50_ (ng/mL)	Elacridar 0.1 μM PAC IC_50_ (ng/mL)	Elacridar 1 μM PAC IC_50_ (ng/mL)
A2780	2.52	2.18	2.50
	(1.69–3.78)	(1.89–2.37)	(1.92–3.33)
	1	1.15 ↓	1.001 ↓
A2780PR1	755	4.66	4.04
	(653–946)	(3.58–6.73)	(3.41–4.99)
	1	162 ↓ **	187 ↓ **
A2780PR2	1970	4.96	4.07
	(1390–3016)	(3.45–6.96)	(3.49–4.52)
	1	397 ↓ **	483 ↓ **

**Table 4:** The summary of cell line resistance to DOX treatment in 2D cell culture conditions in the absence and presence of elacridar at concentrations of 0.1 and 1 μM. The DOX IC_50_ values are indicated for each cell line. For each cell line, the DOX resistance of the lines treated with DOX alone was assigned a value of one. Underlined values indicate multiplicities of sensitivity in the presence of elacridar with respect to the cells treated with DOX alone. The up/down arrows indicate an increase/decrease in IC_50_ value compared to control. * *p* < 0.05 and ** *p* < 0.01.

Cell Line	Control DOX IC_50_ (ng/mL)	Elacridar 0.1 μM DOX IC_50_ (ng/mL)	Elacridar 1 μM DOX IC_50_ (ng/mL)
A2780	22.7	23.3	22.7
	(17.2–33.3)	(18.2–33.6)	(15.0–36.6)
	1	1.03 ↑	1.00
A2780PR1	2033	44.4	50.0
	(886–4328)	(28.6–68.0)	(39.1–69.2)
	1	46 ↓ *	41 ↓ *
A2780PR2	6292	67.8	62.1
	(2631–9420)	(31.6–124)	(43.9–84.8)
	1	92.8 ↓ **	101 ↓ **

**Table 6:** The summary of cell line resistance to PAC treatment in 3D cell culture conditions in the absence and presence of elacridar at a concentration of 1 μM for spheroids formed from 10,000 cells (A) and 20,000 cells (B). The PAC IC_50_ values are indicated for each cell line. The PAC resistance in every cell line treated with PAC alone was assigned a value of one. Underlined values indicate multiplicities of sensitivity/resistance in the presence of elacridar with respect to cells treated with PAC alone. The up/down arrows indicate an increase/decrease in IC_50_, compared to the control.

A			B		
Cell Line	Control PAC IC_50_ (ng/mL)	Elacridar 1 μM PAC IC_50_ (ng/mL)	Cell Line	Control PAC IC_50_ (ng/mL)	Elacridar 1 μM PAC IC_50_ (ng/mL)
A2780	56.7	49.3	A2780	5115	6911
	(40.1–76.7)	(35.5–63.1)		(2636–8746)	(5528–8479)
	1	1.15 ↓		1	1.35 ↑
A2780PR1	13,639	14,069	A2780PR1	15,005	14,552
	(9699–15,713)	(11,855–15,590)		(14,089–16,005)	(14,100–14,837)
	1	1.03 ↑		1	1.03 ↓
A2780PR2	17,053	16,576	A2780PR2	18,517	17,191
	(11,987–21,761)	(13,818–18,660)		(15,124–21,082)	(15,153–18,407)
	1	1.03 ↓		1	1.08 ↓

**Table 7:** The summary of cell line resistance to DOX treatment in 3D cell culture conditions in the absence and presence of elacridar at a concentration of 1 μM, for spheroids formed from 10,000 (A) cells and 20,000 cells (B), respectively. The DOX IC_50_ values are indicated for each cell line. The DOX resistance in the cells treated with DOX alone was assigned a value of one. Underlined values indicate multiplicities of sensitivity/resistance in the presence of elacridar with respect to the cells treated with DOX alone. The up/down arrows indicate an increase/decrease in IC_50_ compared to the control.

A			B		
Cell Line	Control DOX IC_50_ (ng/mL)	Elacridar 1 μM DOX IC_50_ (ng/mL)	Cell Line	Control DOX IC_50_ (ng/mL)	Elacridar 1 μM DOX IC_50_ (ng/mL)
A2780	48.7	41.9	A2780	462	537
	(37.6–59.0)	(22.9–73.5)		(190–679)	(257–664)
	1	1.16		1	1.16 ↑
A2780PR1	>50,000	615	A2780PR1	>50,000	>50,000
		(488–672)			
	1	at least 81.3 ↓		1	1
		p not calculated			
A2780PR2	>50,000	412	A2780PR2	>50,000	>50,000
		(143–733)			
	1	at least 121 ↓		1	1
		p not calculated			

**Table 5:** The summary of cell line resistance to CIS treatment in 2D cell culture conditions in the absence and presence of elacridar at concentrations of 0.1 and 1 μM. The CIS IC_50_ values are indicated for each cell line. The CIS resistance in the cell lines treated with CIS alone was assigned a value of one. Underlined values indicate multiplicities of sensitivity in the presence of elacridar with respect to cells treated with CIS alone. The up arrows indicate an increase in IC_50_ value compared to control.

Cell Line	Control CIS IC_50_ (ng/mL)	Elacridar 0.1 μM CIS IC_50_ (ng/mL)	Elacridar 1 μM CIS IC_50_ (ng/mL)
A2780	3763	4567	4050
	(1785–9117)	(2218–8920)	(1873–7011)
	1	1.21 ↑	1.08 ↑
A2780PR1	7915	9483	9835
	(4784–14,901)	(3357–15,277)	(4730–15,787)
	1	1.20 ↑	1.24 ↑
A2780PR2	6096	7401	7689
	(4013–8889)	(5487–9070)	(4054–9874)
	1	1.21 ↑	1.26 ↑

**Table 8:** Primers used in the Q-PCR reaction.

Transcript	Sequence (5′−3′ Direction) Forward	Sequence (5′−3′ Direction) Reverse	ENST Number http://www.ensembl.org	Product Size (bp)
MDR1	TGACAGCTAC AGCACGGAAG	TCTTCACCTCC AGGCTCAGT	00000265724	131
GAPDH	GAAGGTGAAG GTCGGAGTCA	GACAAGCTTC CCGTTCTCAG	00000229239	199

## Data Availability

The data presented in this study are openly available in [https://repod.icm.edu.pl/dataset.xhtml?persistentId=doi:10.18150/RITKOQ, accessed on 23 January 2025] at [https://doi.org/10.18150/RITKOQ].
